# Public health in Sudan: priorities, challenges, and pathways to resilience in crisis

**DOI:** 10.3389/fpubh.2025.1647917

**Published:** 2025-08-12

**Authors:** Majd A. Alsoukhni, Muna Ibrahim Abdel Aziz, Haytham Qosa, Ibrahim Bani, Maye Omar, Muntasir Mohammed Osman Elhassan, Haitham Bashier Abbas, Yousef Khader, Magid Al-Gunaid, Mohannad Al Nsour

**Affiliations:** ^1^The Eastern Mediterranean Public Health Network (EMPHNET), Amman, Jordan; ^2^Public Health Department, Salford City Council, Salford, United Kingdom; ^3^International Federation of Red Cross and Red Crescent Societies (IFRC) – MENA, Amman, Jordan; ^4^Yale School of Public Health, New Haven, CT, United States; ^5^Nuffield Centre for International Health and Development, University of Leeds, Leeds, United Kingdom; ^6^Republic of Sudan Federal Ministry of Health, Khartoum, Sudan

**Keywords:** Sudan, crisis, impact on health systems, health system resilience, public health

## Abstract

In April 2023, the armed conflict erupted in Sudan, exacerbating the ongoing crisis with widespread violence, health system collapse, and outbreaks of vaccine-preventable, vector-borne, and water-borne diseases. Despite these devastating consequences, the international response has been inadequate, requiring urgent advocacy for increased global support. During the EMPHNET 8th Regional Conference (September 15–18, 2024), a 2-hour forum, “Public Health in Sudan: Priorities and Solutions,” brought together a moderator and six distinguished speakers to discuss critical aspects of the health crisis. The forum addressed four key areas: the health and humanitarian needs in Sudan, strategies for building a resilient health system, the role of multisectoral coordination and integrated policies, and the contributions of local and international actors in crisis response and resource mobilization. Key recommendations emphasized strengthening supply chains, decentralizing resources, and fostering multisectoral collaboration to address health determinants and optimize response efforts. Building health system resilience through training, capacity development, and community-based health solutions was identified as critical. The panel also advocated for sustained, conflict-sensitive funding mechanisms and preventive care to improve public health in Sudan. In conclusion, the discussions highlight the importance of integrating health system resilience into recovery plans and fostering strong partnerships to ensure a health system that is adaptable, inclusive, and sustainable. By addressing immediate needs and preparing for future crises, Sudan can build a robust healthcare system capable of withstanding prolonged challenges.

## Introduction

Before the eruption of the armed conflict in April 2023, Sudan had a long history of persistent instability, resulting in a protracted, complex crisis characterized by violence and displacement (1). By early 2023, Sudan was already experiencing its highest levels of humanitarian need in a decade (2). The conflict in April 2023 further exacerbated this crisis, leading to widespread violence and suffering. The latest estimates declare that the large-scale conflict has killed more than 150,000 people and injured more than 33,000 others (3, 4). As of May 2025, more than 12 million have been forcibly displaced including 8.1 million internally and 3.9 million across borders (5). According to the United Nations High Commissioner for Refugees (UNHCR), Sudan is now facing the largest displacement crisis in the world (6).

The armed conflict has also significantly impacted an already fragile health system in Sudan (7, 8). According to the World Health Organization (WHO) estimates, prior to the conflict, there were 7,300 primary healthcare and 540 public hospitals across the country (9). However, the conflict has led to a collapse of the functioning of health system, exacerbating indirect mortality and morbidity (10). As of March 2025, only 16% of primary healthcare centers, 14% of hospitals, and 40% of sentinel surveillance sites remaining operational, according to Health Cluster data (11).

Many of the health care workforce in Sudan have left the country because of the conflict, while some have been killed. The remaining healthcare workforce are now facing increasingly challenging working conditions (12). Over 100 attacks on healthcare have been verified since the eruption of the conflict, resulting in 183 deaths and 125 injuries (12). These violent incidents have not only resulted in loss of life but also contributed to the severe shortage of qualified healthcare professionals, with only 10% of the pre-conflict ministry of health staff remaining in service (13). As of December 2024, at least 122 health workers have been killed, and 90 arrested, than 100 ambulances and 400 vehicles and trucks have been reported looted, with partial or complete destruction of health institutions and medical supply stores (11, 13).

These conditions have triggered multiple health emergencies compounded by widespread shortages of food, water, shelter, and medical supplies. As of June 2024, the scale of food insecurity and malnutrition in Sudan has reached alarming levels due to the ongoing armed conflict (14). An analysis by the Integrated Food Security Phase Classification (IPC) indicates that Sudan is experiencing the world’s largest hunger crisis, with more than half the population (26 million) facing acute hunger including 755,000 people facing catastrophic conditions (IPC5) (15).

The conflict has contributed to outbreaks of vaccine-preventable, vector-borne, and water-borne diseases, including measles, meningitis, polio, malaria, dengue, and cholera (16, 17). The cumulative number of cholera cases has reached 17,649, and the number of dengue fever cases has reached 194, with 543 deaths caused by cholera and 10 deaths caused by dengue fever, as of October 2024 (18).

Women and child health care services are significantly affected by the ongoing crisis. About 5.8 million (54%) of the IDPs in Sudan are women and girls (19). According to the UN Population Fund, more than 4 million women in Sudan are currently at risk of sexual violence and exploitation (20). Additionally, more than 1.2 million pregnant and breastfeeding women, with thousands lacking access to basic maternal health services (19). The conflict has significantly affected children, with millions fleeing their homes and being displaced both within the country and across its borders. Nearly 14 million children are in need of humanitarian assistance (21).

Despite all the aforementioned consequences of the devastating armed conflict, the response from the international community has remained significantly inadequate (22, 23). The United Nations’ funding goal of $2.7 billion for 2024, only 48% has been met (24). The urgent humanitarian needs were also underscored by EMPHNET Public Health Emergency Management center when a team of experts visited Sudan in May 2024. The visit revealed a huge gap in humanitarian support and that the situation is far worse than described in the media. Therefore, there is an urgent need to advocate more effectively for Sudan and to draw the attention of the international community toward providing greater support.

The forum on “Public Health in Sudan: Priorities and Solutions” aimed to shed light on the health situation in Sudan to raise international awareness about the health and humanitarian crisis and strengthen advocacy for increased support. The forum was held during the 8th Regional Conference of the Eastern Mediterranean Public Health Network (EMPHNET), from September 15–18, 2024. By bringing together diverse stakeholders, the forum sought to unify collective efforts to provide immediate relief, rebuild the health infrastructure, and ensure a resilient health system for the future. This viewpoint aimed to analyze the key healthcare challenges facing Sudan amidst the ongoing crisis, explore approaches for enhancing the resilience of Sudan’s health system, and examine the importance of coordinated efforts across sectors in crisis response.

## Forum description

The 2 h forum provided a platform for international experts to address the challenges and opportunities for strengthening Sudan’s health system amid the ongoing conflict. Moderated by an Associate Professor from Yale School of Public Health, the forum began with an introduction that set the stage for an in-depth exploration of Sudan’s health crisis. The panel featured six distinguished speakers, each bringing their unique perspectives and expertise to the discussion. The first speaker, Head of the Health Emergency and Epidemic Control (HEEC) department at the Sudan Federal Ministry of Health, provided an overview of the current health situation in Sudan, highlighting the pressing humanitarian needs and priorities that must be addressed.

An Associate Professor at the University of Leeds, discussed strategies for rebuilding a resilient health system, focusing on the importance of sustainable interventions in the face of ongoing challenges. Then, the Director of Public Health in Salford, UK, shared insights on the necessity of multisectoral coordination in health policies to ensure comprehensive support for Sudan’s healthcare needs.

The forum also featured contributions from a Public Health Consultant/Medical Epidemiologist at the Ministry of Public Health in Qatar, who highlighted the vital role of the Sudanese diaspora in supporting efforts during the crisis, and the Head of the Health and Care team at The International Federation of Red Cross and Red Crescent Societies (IFRC) -MENA, who discussed the essential involvement of the Red Crescent Society and local actors in crisis response.

To conclude the session, the Regional Emergency Director for WHO EMRO, reflected on the insights shared during the forum, emphasizing WHO’s critical role in coordinating efforts to address the health crisis in Sudan. The forum not only facilitated meaningful dialogue among experts but also reinforced the commitment to mobilizing resources and strategies to improve public health in Sudan.

## Key themes

The forum’s discussions revolved around four main areas critical to addressing the current health crisis. Those areas included the current health crisis and humanitarian needs in Sudan, strategies for building a resilient and sustainable health system capable of withstanding prolonged crises, the importance of multisectoral coordination and integrated health policies to ensure comprehensive support for healthcare needs, and finally, the essential roles of local and international actors in crisis response and resource mobilization. These focal points provide a comprehensive framework for understanding the paths to improve public health in Sudan despite the challenging situation.

## Current health crisis and humanitarian needs in Sudan

The ongoing conflict has destroyed Sudan’s already fragile health system, leaving it in a state of near-total collapse. “*The war has led to the near-complete destruction of health services*,” *said the Head of HEEC department at Sudan FMOH*. Within the first 2 weeks of fighting in Khartoum, 60% of health facilities were forced to close. After 6 months of sustained conflict, 70% of health facilities across all conflict-affected areas are non-functional. Critical health infrastructure, such as the cold chain for vaccines, has been disrupted, and vital transport for health services has been severely diminished, with over 100 ambulances and 400 vehicles lost.

The ongoing war has led to multiple disease outbreaks with high mortality rates attributed to cholera due to shortages in medical supplies. The resurgence of diseases like measles and polio, particularly in hard-to-reach areas, poses additional threats to public health. As of February 2025, more than 8.8 million people were internally displaced within Sudan, and over 12.3 million were displaced overall, including those who fled the country (25). A significant proportion of this movement has occurred from urban centers, such as Khartoum, Omdurman, and Bahri, to rural and peripheral areas (26). The massive displacement from urban centers to rural areas has strained already limited health resources, leading to a brain drain that leaves remaining health workers overwhelmed (27).

Beyond human resources, this large-scale displacement has impacted multiple components of Sudan’s health system. Rural and host communities, often lacking adequate health infrastructure, have been overwhelmed by the sudden surge in demand for services (28). Service delivery has been disrupted in both origin and destination areas, with rural health facilities facing overcrowding, medicine shortages, and inadequate equipment (27). The health information system (HIS) has struggled to track health indicators and coordinate care due to the mobility of populations and closure of reporting sites (29). The supply chain has also suffered, with the centralization of medical stocks in Khartoum creating bottlenecks in reaching remote areas (29).

Despite these challenges, the displacement has also presented a potential opportunity to reshape service delivery models. The increased demand in outlying areas has underscored the need to expand services beyond urban hubs and invest in rural health infrastructure. This shift could support a more resilient, decentralized health system capable of responding more effectively in times of crisis (30).

“*Despite these challenges, the resilience of Sudan’s health workforce remains a beacon of hope*,” *said the Head of HEEC department.* The Federal Ministry of Health and other national and international partners have concentrated efforts on maintaining emergency essential healthcare services and epidemic control. In response to the ongoing crisis, the following top five priorities have been determined for the health sector:

1. Ensuring the supply of essential and life-saving medicines and medical consumables
2. Ensuring continuity of emergency basic and essential health services in hospitals and health institutions
3. Enabling the health system to control epidemics and reduce mortalities and morbidities
4. Ensuring the continuity of maternal and child health programs
5. Commanding a coordinated response with UN agencies, INGOs, and health sector partners

Response to the crisis in Sudan has unfolded in phases; there was a critical shortage of life-saving supplies, with national stocks nearly depleted. Within a few months, redistribution efforts and donor support began to stabilize essential supply chains, achieving about 50–70% availability through decentralizing regional hubs.

Essential healthcare services faced a similar trajectory. Many facilities shut down as health staff struggled with uncertainty, but state-level capacities were strengthened with national support teams and redeployed specialists. Now, roughly 75% of facilities are operational, with some introducing specialized services that were previously unavailable.

Epidemic control efforts initially struggled due to dysfunctional emergency response systems, resulting in widespread outbreaks. Though epidemic curves have since stabilized, significant risks remain. Maternal and child health (MCH) services also faced setbacks, with preventable diseases like measles spreading in 10 states. Vaccines eventually reached all states, resuming first-dose vaccinations after a one-year gap in some States, although operational challenges persist.

Coordinated efforts with UN agencies and I/NGOs encountered complex security issues. Health staff initially considered fleeing due to safety concerns, but a coordinated cluster forum has now been established, improving cooperation across the health sector and enhancing Sudan’s capacity to respond effectively. Each phase reflects a gradual strengthening of Sudan’s health system, driven by resilience and strategic support amidst ongoing conflict.

The centralization of medical supplies in Khartoum left much of the health system vulnerable when the capital was impacted by the conflict. “*We have learned some crucial lessons from this ongoing crisis. Decentralizing the health system and securing supplies outside the capital are essential for future resilience*,” said *the Head of HEEC department*.

## Building resilient and sustainable health system

The WHO define the health system recovery as the rebuilding, restoration and improvement of the health system’s components and core public health functions, in alignment with the principles of build back better and sustainable development (31). “*Rebuilding Sudan’s health system is a critical mission requiring a balance between humanitarian assistance and addressing emerging health needs*,” said the Associate Professor from the University of Leeds. The current landscape in Sudan is marked by ongoing conflict, natural disasters such as floods, and a resurgence of diseases. The panelists emphasized the necessity of building a resilient health system capable of preparing for and responding to future crises.

After the acute phase of response to an emergency is over, the health system must be restored or completely rebuilt. This represents an opportunity to create a more resilient and fit-for-purpose system that promotes and safeguards population health and health security. “*Resilience is an ability, not an outcome. Strengthening the capacity of health systems to be resilient is critical to continue delivering essential preventive and curative healthcare services during crises,” said* the Associate Professor from the University of Leeds.

The WHO health systems framework identifies six core components essential for strengthening health systems: service delivery, health workforce, health information, medical products, vaccines and technologies, financing, and leadership/governance (32). These components are interrelated and necessary for creating a resilient health system that ensures quality care, efficient resource use, and effective management to meet population health needs (33).

Building on the WHO health systems framework, theories and frameworks of health systems strengthening and resilience emphasize the importance of three key levels of capacity: absorptive, adaptive, and transformative (34, 35). Absorptive capacity focuses on a health system’s ability to absorb shocks and manage routine health needs. Adaptive capacity refers to the system’s flexibility to respond to changing circumstances and emerging challenges, while transformative capacity highlights the system’s ability to undergo fundamental changes that improve long-term health outcomes (35). These capacities work in tandem to ensure that health systems are not only capable of responding to crises but are also able to evolve and thrive in the face of future challenges (36).

To achieve resilience for Sudan, a comprehensive reorientation of the health system is necessary, focusing on redefining essential health services, enhancing emergency response capacity, and ensuring sustainable, coordinated actions among diverse stakeholders (37). The panelists emphasized that this reorientation must be tailored to the unique characteristics and needs of different localities in Sudan. It involves redefining the essential health service package, building emergency response capacity, reskilling and empowering the health workforce, and coordinating efforts across actors and resources to establish a sustainable, resilient health system. Rebuilding efforts must foster meaningful, sustained action to address the health crises exacerbated by ongoing conflict. It is crucial to unite diverse stakeholders to provide immediate relief, rebuild health infrastructure, and lay the foundation for a resilient health system capable of facing future challenges.

## Multisectoral coordination and policy integration

Health outcomes in conflict-affected regions of Sudan are influenced by a range of factors, relying not only on the healthcare system but also significantly on community actions and resilience (38). Achieving improved health outcomes requires a decentralized, sustainable approach, where collaboration across sectors, such as education, economy, diplomacy, and the community, plays a central role in strengthening the health system (39). As highlighted by the Director of Public Health in Salford, “*Drawing from lessons learned during the COVID-19 pandemic, it is evident that solutions already exist, but effective coordination is critical to success*.”

Multisectoral coordination for health in Sudan requires the active engagement of both national and international organizations, guided by the philosophy of “*starting where you are, using what you have, and doing what you can.*” This emphasizes the need for both individual and collective efforts to address Sudan’s complex health challenges. The panelists stressed that by fostering new partnerships and integrating efforts across various sectors, Sudan can more effectively tackle its health and social challenges. “*The success of multisectoral coordination should be measured by the effectiveness of partnerships and accessibility of health services. Small collective actions can lead to significant progress. Resilience and unity are key to addressing Sudan’s health challenges, highlighting the need for sustained, coordinated efforts across sectors for a healthier future,*” said the Director of Public Health in Salford.

The concept of “*thriving communities*” was also highlighted as a key strategy for addressing Sudan’s ongoing health challenges. This concept emphasizes providing access to preventive care and support before more intensive healthcare needs arise (40). In the context of Sudan, where the healthcare system is under significant strain due to conflict and limited resources, a holistic, community-centered approach is essential to tackle health and development challenges. This approach prioritizes preventive care, community engagement, and robust social support systems where individuals to maintain good health and manage risks before they escalate into more severe conditions (41). By fostering thriving communities, Sudan can ensure accessible health resources, promote wellness, build resilience, and reduce dependence on resource-intensive healthcare interventions, thus making a significant step toward long-term health improvements despite ongoing challenges.

## Engagement of local and international actors in crisis response

The response to Sudan’s ongoing public health crisis relies heavily on both local actors and international organizations working together to address urgent health and humanitarian needs. The Sudanese diaspora, with over 2.1 million individuals abroad (42), has played an instrumental role in providing financial, medical, and social support during the conflict. “*Through partnerships with community organizations and Sudan Federal Ministry of Health, the Sudanese Doctors Association in Qatar (SUDAQ) have contributed to* var*ious initiatives, such as sending essential medical equipment, offering healthcare expertise, and advocating to raise awareness of the crisis*,” said the Public Health Consultant at the Ministry of Public Health in Qatar. Their support extends to sustaining dialysis units, rehabilitating children affected by the conflict, and aiding displaced individuals with basic necessities like water and shelter. The diaspora’s commitment to education and healthcare continuity is evident in their support of over 2,100 Sudanese medical students in Qatar.

The Sudanese Red Crescent Society (SRCS) plays a significant role in local humanitarian efforts, *“Since the escalation of the conflict in April 2023, SRCS has reached over 124,000 people with WASH services, 150,000 with food aid, and 100,000 with essential health services,”* said the Head of the Health and Care team at IFRC-MENA. Despite challenges like limited funding and the politicization of humanitarian aid, SRCS has continued operations, providing critical support in health, disease prevention, water, sanitation, shelter, and psychosocial care. They have also collaborated with the International Committee of the Red Cross (ICRC) on family tracing efforts for displaced families. *“Localization is key to building the resilience of communities and healthcare systems, and SRCS is a prime example of this,”* emphasized the Head of the Health and Care team at IFRC-MENA, highlighting the importance of tailoring aid to meet specific community needs effectively.

International organizations, particularly the World Health Organization (WHO), have also provided critical support and coordination (43). The Regional Emergency Director for WHO EMRO underscored the organization’s commitment to Sudan, despite global attention being focused on other crises. *“Sudan’s crisis remains largely neglected, and bringing attention to this forgotten crisis is one of WHO’s key roles,”* he noted. WHO’s approach includes co-leading the health cluster with the Ministry of Health, coordinating over 80 health cluster members to ensure a unified response. Their work spans national and sub-national levels, including cross-border operations in countries hosting Sudanese refugees. WHO has provided direct support to 22 hospitals in 11 states, numerous primary healthcare facilities, and 122 stabilization centers for acute malnutrition (44). Additional initiatives include a cholera vaccination campaign to address disease outbreaks, with WHO partnering with the World Bank and UNICEF to deliver comprehensive healthcare services, with 404,000 doses of oral cholera vaccine have been administered (45, 46).

Despite these combined efforts, over 50% of health services in Sudan remain inaccessible due to infrastructure damage and funding shortages. *“Funding is always a concern, as our association is nonprofit and relies on member contributions. However, we remain optimistic and committed,”* said the Public Health Consultant at the Ministry of Public Health in Qatar. The Head of the Health and Care team at IFRC-MENA added, *“When the conflict erupted in Sudan, we launched an appeal for $60 million but received less than $6 million due to the politicization of humanitarian funding.”*

Both SRCS and WHO continue to operate under challenging conditions, underscoring the resilience and dedication of local and international actors in supporting Sudan through this humanitarian crisis. The Regional Emergency Director for WHO EMRO commended the dedication of Sudanese health workers, noting, *“I’ve always been impressed by the strength of the health system and the dedication of Sudanese health workers.”* Together, the coordinated efforts of the diaspora, local organizations, and international bodies exemplify the power of partnerships in building resilience and addressing Sudan’s pressing public health challenges.

## Conclusion

In conclusion, Sudan’s health sector has faced immense challenges due to ongoing conflict, infrastructure damage, and a critical shortage of resources. The Federal Ministry of Health, along with national and international partners, has focused on maintaining essential healthcare services, epidemic control, and supporting emergency response efforts. Through the implementation of five key priorities, including ensuring the availability of life-saving medical supplies and fostering multisectoral partnerships, significant strides have been made in stabilizing and gradually rebuilding the health system.

Despite these efforts, more than half of the health services in Sudan remain inaccessible, with many facilities struggling to resume full operations. The ongoing support from the Sudanese diaspora, local and international organizations highlight the essential role of collaboration in responding to the crisis. Their collective contributions, from supplying medical equipment to coordinating health clusters, demonstrate the power of coordinated, sustained action.

Finally, building a resilient and sustainable health system in Sudan is critical. This will require a decentralized approach to prevent future disruptions, strengthened emergency response capacities, and a focus on preventive care and community-based health solutions. By integrating health system resilience into recovery plans and fostering strong, multisectoral partnerships, Sudan can better prepare for future crises, aiming for a health system that not only meets immediate needs but is adaptable, inclusive, and sustainable.

## Recommendations

The key recommendations from the panel discussion emphasized the need to strengthen supply chains and decentralize resources to ensure uninterrupted access to life-saving medicines and consumables during conflicts. Multisectoral collaboration and policy integration were also highlighted as essential to addressing health determinants, streamlining resources, and optimizing response efforts. Building health system resilience through training and capacity development was identified as crucial to ensuring the system’s absorptive, adaptive, and transformative capacities. Furthermore, the panel underscored the importance of promoting community engagement and local leadership in health initiatives to foster trust and implement culturally relevant solutions, such as mobilizing communities for public health campaigns, preventive health education, and outreach, particularly in underserved areas. Lastly, the discussion called for advocating depoliticized, sustained conflict-sensitive funding that extends beyond emergency aid through the implementation of transparent mechanisms for fund allocation, aimed at building donor trust and securing ongoing support.

## Data Availability

The raw data supporting the conclusions of this article will be made available by the authors, without undue reservation.
